# Monthly pulse methylprednisolone infusions in patients with non-idiopathic pulmonary fibrosis interstitial lung diseases: a single-center retrospective analyses

**DOI:** 10.1177/17534666251342661

**Published:** 2025-05-30

**Authors:** Dean Kellogg, Jay Peters, Jesse Sherratt, Sebastian Ocrospoma Heraud, Fatima Dollar, Anoop M. Nambiar

**Affiliations:** South Texas Veterans Health Care System, 7400 Merton Minter Drive, San Antonio, TX 78229, USA; University of Texas Health San Antonio, San Antonio, TX, USA; University of Texas Health San Antonio, San Antonio, TX, USA; South Texas Veterans Health Care System, San Antonio, TX, USA; University of Texas Health San Antonio, San Antonio, TX, USA; South Texas Veterans Health Care System, San Antonio, TX, USA; University of Texas Health San Antonio, San Antonio, TX, USA; South Texas Veterans Health Care System, San Antonio, TX, USA; University of Texas Health San Antonio, San Antonio, TX, USA; South Texas Veterans Health Care System, San Antonio, TX, USA; University of Texas Health San Antonio, San Antonio, TX, USA; South Texas Veterans Health Care System, San Antonio, TX, USA

**Keywords:** corticosteroids, non-IPF, interstitial lung disease, progressive pulmonary fibrosis, pulse corticosteroids

## Abstract

**Background:**

Non-idiopathic pulmonary fibrosis interstitial lung diseases (non-IPF ILDs) comprise a broad spectrum of pathologies with varying degrees of inflammation and fibrosis. Progressive fibrosing ILD is associated with significant mortality and limited treatment options. Standard regimens employ multimodal immunosuppression, most commonly prolonged courses of oral corticosteroids (OCS), that are associated with a high risk of adverse effects and limited proven efficacy.

**Objectives:**

This study investigates the safety, tolerability, and effectiveness of monthly intravenous pulse methylprednisolone (PMP) for the treatment of patients with progressive non-IPF ILD.

**Design:**

Retrospective single-center cohort study of patients at an academic tertiary referral center for ILD between October 2019 and September 2022.

**Methods:**

All non-IPF ILD patients who received intravenous PMP (1000 mg daily for three consecutive days/month) between October 2019 and September 2022 were included. The decision to treat was based on a multidisciplinary consensus diagnosis following ATS/ERS/JRS/ALAT guidelines and confirmed or at high risk for ILD progression. Treatment continuation was contingent upon pulmonary function test (PFT) improvement (assessed approximately every 3 months), tolerable adverse events, and shared decision making with patients. Effectiveness was measured by a change in forced vital capacity (FVC) and diffusion limit of carbon monoxide (DLCO), with improvement being defined as an absolute increase in either FVC >5% or DLCO >10% from baseline.

**Results:**

Thirty-three patients received PMP at our center. One patient died of an acute exacerbation of ILD. Of the 32 patients included for analysis, 17 (53%) exhibited improved lung function with PMP between PFTs, which was maintained for a median follow-up of 209 days. The regimen was generally well-tolerated, with the most common adverse effects being insomnia and restlessness on infusion days. Advanced disease, indicated by lower FVC, traction bronchiectasis, and oxygen dependence, predicted poor response.

**Conclusions:**

PMP may offer a safer, better-tolerated, and more effective treatment for progressive non-IPF ILD than prolonged OCS. Notably, a third of fibrotic hypersensitivity pneumonitis patients showed improved FVC after 3 months of PMP, defying expectations of steroid non-responsiveness. However, further well-designed controlled prospective clinical trials are needed to confirm our findings and establish long-term safety.

## Plain language summary

Pulse Methylprednisolone for Progressive Non-IPF ILD - a potential alternative to oral steroids

Non-Idiopathic Pulmonary Fibrosis interstitial lung diseases (non-IPF ILDs) are a broad group of diseases with inflammation and scarring of the lungs. Current treatment centers on oral corticosteroids (primarily prolonged prednisone) despite limited evidence and significant adverse events. This retrospective study reports our experience with using monthly pulse methylprednisolone (PMP) and contributes to the search for identifying treatable traits in non-IPF ILDs. 53% of the patients who received PMP had significant improvement in ILD, and there were well tolerate side- effects during treatments. We also identify traction bronchiectasis and home oxygen use as negative prognostic factor for response to steroids. This pilot study lays the foundation for future trials of pulse steroids in non-IPF ILDs as a potential alternative to prolonged oral corticosteroids.

## Background

Interstitial lung diseases (ILDs) represent a diverse group of pulmonary disorders characterized by inflammatory and fibrotic changes in the lung parenchyma.^1^ Conditions classified as non-idiopathic pulmonary fibrosis ILD (non-IPF ILD) include hypersensitivity pneumonitis (HP), connective tissue disease-related ILD (CTD-ILD), and other idiopathic interstitial pneumonias (IIPs), excluding IPF.^2^ Disease trajectory is highly variable and patients with non-IPF ILD may develop a progressive fibrosing phenotype called progressive pulmonary fibrosis (PPF) defined by worsening respiratory symptoms, declining lung function as measured by forced vital capacity (FVC) and diffusing capacity of the lungs for carbon monoxide (DLCO), increased radiologic fibrosing abnormalities, worse health-related quality of life, and premature death. Despite early recognition and interventions, approximately 30% of non-IPF ILD patients develop PPF, which is associated with increased morbidity and mortality rates similar to IPF.^2^

Current standard-of-care for non-IPF ILD focuses on immunosuppression, which has traditionally relied on oral systemic corticosteroids (OCS), such as prednisone, prednisolone and dexamethasone, particularly when inflammatory features are present (e.g., ground-glass opacities (GGOs), 20%–40% BAL lymphocytosis, granulomas, elevated C-reactive protein (CRP)).^3,4^ International guidelines recommend systemic corticosteroids for acute exacerbation of IPF, based on very low-quality evidence,^5^ but no such recommendation exists for non-IPF ILD, especially those with PPF. In fibrotic HP and pneumoconiosis, OCS is the only reported treatment, but does not improve long-term outcomes.^6^ Moreover, the effectiveness of OCS varies widely, with most studies reporting modest short-term improvements in half of non-IPF ILD patients. In fibrotic nonspecific interstitial pneumonia (NSIP) receiving OCS with or without cytotoxic agents, 53% of patients improved at 1 year, and those that progressed suffered high mortality (69% at 5 years).^7^ One study demonstrated transient improvement in acute respiratory distress without survival benefit with high-dose prednisolone taper after an AE-IIP.^8^ The largest study to show the benefit of OCS was a retrospective study of HP patients without extensive fibrosis which demonstrated durable survival benefit to 24 months.^9^

Reported clinical outcomes also vary with dosing and type of OCS. Prednisone (0.5–1 mg/kg predicted body weight per day) is widely used based on observational data and expert opinion with highly variable regimens across small clinical trials.^10^ However, evidence is limited to only one placebo-controlled randomized controlled trial (RCT).^11^ In addition, long-term prednisone use is associated with several significant adverse events (AEs), including osteoporosis, hypertension, pneumonia, and neuropsychiatric disturbances.^12,13^ Another study of acute exacerbations of ILD showed improved survival with high-dose prednisone (>1 mg/kg).^14^ Other regimens for acute exacerbations or rapidly progressive non-IPF ILDs included 500 mg of IV methylprednisolone for 3 days, but did not show improved mortality.^15^ The uncertainties surrounding corticosteroids in non-IPF ILDs underscore the need for treatment alternatives. High-dose methylprednisolone has been reported as a potential treatment for a variety of diseases, such as allergic bronchopulmonary aspergillosis (ABPA),^16^ multiple sclerosis,^17^ and chronic inflammatory demyelinating polyradiculoneuropathy.^18^ However, it is an established treatment for acute exacerbations of both IPF and non-IPF ILD,^14,19^ pulse systemic corticosteroid regimens in ILD have not been studied in the treatment of patients with non-IPF ILD.^20^

This retrospective study investigates the safety, tolerability, and effectiveness of a 3-day monthly pulsed regimen of high-dose (1000 mg) IV methylprednisolone (PMP) in non-IPF ILD patients with confirmed or at high risk of progression.

## Methods

### Study design and participants

This retrospective single-center cohort study analyzed prospectively generated data from patients at an academic tertiary referral center ILD with a Pulmonary Fibrosis Foundation Center of Excellence designation. Patients were discussed at our multidisciplinary discussion (MDD) between October 2019 and September 2022, and there was consensus that systemic corticosteroids were indicated to either stop or prevent the progression of ILD. This comprehensive approach included careful consideration of history, physical exam, comorbidities, environmental exposures, autoimmune and HP serologies, high-resolution CT scan (HRCT), and pathology results when available by a multidisciplinary team of ILD pulmonologists, lung transplant pulmonologists, an ILD pathologist, and thoracic radiologist. ATS/ERS/JRS/ALAT guidelines for diagnosis of ILD were followed.^21,22^ Fibrotic HP was differentiated from non-fibrotic HP by radiologic evidence of fibrosis suggested by peripheral reticulation, traction bronchiectasis, or honeycombing on HRCT. ILD consensus diagnosis allowed for multiple diagnoses (e.g., the same patient may have organizing pneumonia and CTD-ILD). The reporting of this study conforms to the Strengthening the Reporting of Observational Studies in Epidemiology (STROBE) statement.^23^

Background immunosuppression with either mycophenolate mofetil, azathioprine, or rituximab was started at the time of initiating PMP or subsequently within 1–3 months if there was a significantly positive response to PMP. Pneumocystis prophylaxis was typically with trimethoprim-sulfamethoxazole 160–800 mg three times per week (TIW) unless contraindicated. Patients with HP were also managed with professional environmental antigen eviction when appropriate.

### Intervention

The PMP regimen consisted of 1000 mg of intravenous methylprednisolone sodium succinate daily for three consecutive days per month, administered for a minimum of 3 months. Treatment was continued for an additional three or more months only if no significant AEs and if the absolute FVC percent predicted based on the NHANES III database (%pp) increased by 5% or greater or if the absolute DLCO %pp increased by 10% or greater from baseline. For patients who stabilized on treatment, the decision to maintain monthly PMP was based on shared decision making with the patient.

This study included all patients who started the PMP and did not exclude patients.

### Assessment and outcomes

The effectiveness of the PMP regimen was assessed by longitudinal PFT changes. Improvement was defined as an increase in FVC >5% or DLCO >10% compared to baseline prior to initiation of PMP, and worsening was defined as a decrease in FVC >5% or DLCO >10%. All PFT data were sourced from routine clinical documentation. Radiographic data were acquired from MDD documentation by a thoracic radiologist.

Safety and tolerability were assessed through a comprehensive review of AEs, as documented during nursing visits and routine pulmonary clinic visits, or by changes in laboratory data. Serious AEs were also noted, including hospitalizations and deaths. Patients were weighed at every visit to assess for possible weight gain.

### Statistical analysis

Continuous variables are presented as means and standard deviations, while categorical variables are shown as frequencies and percentages. Comparisons between patients who improved and those who did not were made using Student’s *t*-test for continuous variables and Fisher’s exact test for categorical variables, as appropriate. Statistical significance was defined as *p* < 0.05.

For the primary outcome, longitudinal changes in pulmonary function were assessed by FVC and diffusing capacity for carbon monoxide (DLCO) from baseline to follow-up at 3, 6, and 9 months. Differences in FVC and DLCO over time were analyzed using a repeated measures analysis of variance (ANOVA). Kaplan–Meier survival analysis was used to estimate time to ⩾5% FVC decline, and comparisons between groups (e.g., presence of traction bronchiectasis or home oxygen use) were assessed using the log-rank test. Logistic regression analysis was employed to identify predictors of non-improvement, with odds ratios and 95% confidence intervals calculated for each covariate. All statistical analyses were performed using SPSS version 28.0 (IBM Corp., Armonk, NY, USA).

## Results

The study cohort included 33 non-IPF ILD patients who received PMP, with a median follow-up of 209 days. Of these, 17 patients (53%) showed improvement as defined as an increase in FVC >5% or DLCO >10%, whereas 15 patients (47%) did not improve, including 1 death.

### Patient demographics and clinical characteristics

The demographic characteristics are summarized in Table 1. The mean age of the cohort was 67.0 years. The mean age and distribution of sex were similar between patients who improved with PMP versus those who did not improve (53% female). Patients who did improve had a higher mean weight (94.4 kg vs 83.8 kg) and higher body mass index (33.4 kg/m^2^ vs 31.2 kg/m^2^), although these parameters did not reach statistical significance. Comorbidities were prevalent in both groups, with hypertension and a history of smoking being the most common.

**Table 1.** Demographics.

Variable	Overall	Improved	Not improved
*n*	33	17	15
Female	17	9	8
Male	16	8	7
Age (years)	67.0	65.5	68.7
Ethnicity (*n*)			
White	21	12	9
Hispanic	10	4	5
Weight (kg)	89.3	94.4	83.8
BMI (kg/m^2^)	32.2	33.4	31.2
Comorbidities			
Hypertension	12	4	7
Former smoker	13	6	7
Active smoker	0	0	0
Asthma	8	4	4
History of malignancy	5	4	1
COPD	4	2	2
Diabetes	3	1	2
Heart failure	0	0	0
Immune deficiency	0	0	0

Mean unless otherwise specified; One death excluded from improved versus not improved.

BMI: body mass index; COPD, chronic obstructive pulmonary disease.

The entire cohort comprised patients with moderate ILD based on baseline FVC 56.6% and DLCO 43.1% predicted. The presence of CTD-ILD varied between groups, with rheumatoid arthritics ILD (RA-ILD) more prevalent in the patients who did not improve with PMP (*n* = 6 vs 1; Table S1). The spectrum of inciting antigens in patients with HP included mold and bird antigens, among others, but was not associated with improvement with PMP (Table S2).

### Pulmonary function and radiographic features

**Figure 1.**
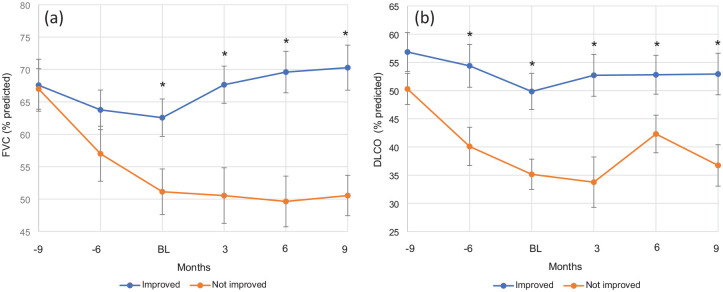
Impact of PMP on ILD progression. Longitudinal changes in (a) forced vital capacity (percent predicted) and (b) diffusing capacity for carbon monoxide (percent predicted). Groups based on clinical improvement (“Improved”) or lack thereof (“Not Improved”). Data points are representative of PFTs performed immediately prior to PMP (BL) and rough intervals (6 and 9 months before PMP and 3, 6, and *9* months afterward). *p < 0.05 improved vs not improved group. BL, baseline; ILD, interstitial lung disease; PMP, pulsed methylprednisolone sodium succinate.

Non-IPF ILD patients that improved with PMP had milder baseline disease as demonstrated by better baseline pulmonary function based on %FVC (62.6 vs 51.1, *p* = 0.02), %DLCO (49.8 vs 35.1, *p* = 0.002) and lower home oxygen use (41% vs 87%; Table 2). Longitudinal PFT data (Figure 1) showed significant improvement in both FVC and DLCO in the improved group that persisted to approximately 9 months after PMP initiation. Specifically, the mean FVC increased from baseline values, showing statistically significant improvement at 3-, 6-, and 9-months post-treatment (*p* < 0.05 at each interval). The “not improved” group experienced a more rapid decline prior to PMP initiation but stabilized after starting treatment.

**Table 2.** ILD characteristics.

Variable	Overall	Improved	Not improved	*p*-Value
ILD duration (years)	2.22	2.33	2.15	
Home oxygen use (*n*)	21 (64%)	7	13	
Baseline PFT				
% FVC	56.6	62.6	51.1	0.02
% DLCO	43.1	49.8	35.1	0.002
CTD	12	5	7	
HP	19	7	11	
Fibrotic HP	12	4	8	
Non-fibrotic HP	6	3	3	
Cryptogenic OP	1	1	0	
Idiopathic NSIP	3	2	1	
IPAF	2	2	0	
Sarcoidosis	1	0	1	
Unclassifiable	2	2	0	
Other	2	1	1	
Treatments				
Methylprednisolone				
Cumulative dose (g)	22.8	26.1	20.6	0.30
Duration (months)	7.6	7.9	8.1	
Mycophenolate	27	14	12	
Azathioprine	2	1	1	
Rituximab	2	2	0	
Nintedanib	6	2	4	

CTD, connective tissue disease; DLCO, diffusion limit of carbon monoxide; FVC, forced vital capacity; HP, hypersensitivity pneumonitis; ILD, interstitial lung disease; IPAF, interstitial pneumonia with autoimmune features; NSIP, nonspecific interstitial pneumonia; OP, organizing pneumonia.

### Treatment regimen characteristics

The median duration of PMP treatment was similar between the groups (approximately 7.6 months; Table 2). As continuation was contingent on improvement, the cumulative dose of methylprednisolone was higher in the improved group (26.1 g vs 20.6 g), albeit not reaching statistical significance (*p* = 0.30). Concomitant immunosuppressive therapy, predominantly with mycophenolate, was used in most patients, with no significant difference between the groups. The reasons for discontinuation are summarized in Table S3, with the most common being a plateau in pulmonary function.

**Figure 2.**
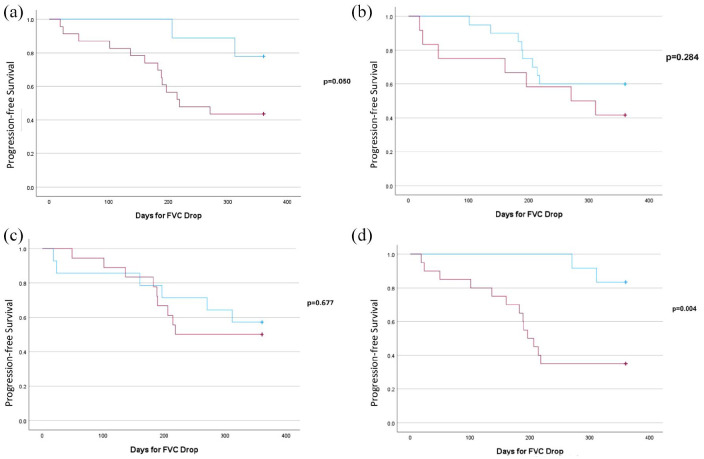
Kaplan–Meier plot of decline in FVC by >5%. (a) Traction bronchiectasis: patients with traction bronchiectasis (red) showed a faster decline in FVC compared to those without (blue; *p* = 0.05). (b) CTD-ILD: no significant difference in time to FVC decline between patients with CTD-ILD (red) and those without (blue; *p* = 0.284). (c) HP: no significant difference in time to FVC decline between patients with HP (red) and those without (blue; *p* = 0.677). (d) Home oxygen use: patients requiring home oxygen (red) experienced a significantly faster decline in FVC compared to those not requiring it (blue; *p* = 0.004). CTD-ILD, connective tissue disease-related interstitial lung disease; FVC, forced vital capacity; HP, hypersensitivity pneumonitis.

Presumed steroid-responsive traits are shown in Table 3. Patients with higher baseline CRP (12.2 mg/L vs 3.6 mg/L) and mild patchy GGOs on HRCT were more likely to show improvement with PMP treatment. Patients with radiologic traction bronchiectasis on baseline HRCT had poorer response to PMP, progressed significantly faster (Figure 2(a)), and were less likely to improve with PMP (OR 12.205, *p* = 0.03; Table 4). In addition, the use of home oxygen was significantly associated with a lack of improvement in patients with non-IPF ILD treated with PMP (Figure 2(d)). Neither the presence of CTD-ILD nor HP showed a statistically significant impact on the rate of FVC decline.

**Table 3.** Baseline treatable traits by group.

Variable	Improved	Not improved
Radiographic features, *n* (%)		
GGO	14 (82)	10 (67)
Mild, patchy	8 (47)	3 (20)
Diffuse	6 (35)	7 (47)
Mosaicism	3 (18)	4 (27)
Traction bronchiectasis	9 (53)	14 (93)
Honeycombing	2 (12)	6 (40)
Inflammatory markers		
CRP (mg/L)	12.2 [7.0–17.3]	3.6 [3.0–6.8]
ESR (mm/h)	17 [13.3–28.8]	19 [11.3–31.0]
Monocyte (k/µL)	0.58 [0.4–0.73]	0.48 [0.4–0.6]
Lymphocyte (k/µL)	1.58 [0.8–2.2]	1.7 [1.3–2.2]

Median [IQR].

CRP, C-reactive protein; ESR, erthrocyte sedementation rate; GGO, ground-glass opacities.

**Table 4.** Logistic regression for no improvement.

Covariate	Odds ratio	95% CI	*p*-Value
Gender (F)	0.45	0.04–4.89	0.51
BMI	1.77	0.82–3.83	0.14
Prior smoker	2.91	0.30–28.63	0.36
CTD-ILD	5.42	0.45–64.97	0.18
Traction bronchiectasis	12.21	1.23–121.56	0.03
Honeycombing	1.22	0.08–19.20	0.89

Fisher’s exact test, one-sided.

BMI, body mass index; CTD-ILD, connective tissue disease-related interstitial lung disease.

### Safety and tolerability

AEs were generally manageable; most common were insomnia (21%) and restlessness (15%) reported on the day of infusion (Table 5), which often improved and resolved after 7 days. Three patients (9%) experienced mild weight gain (5–10 pounds) while on treatment. No significant changes were observed in other metabolic, psychiatric, or general health parameters. There were no differences in AE incidence between the improved and not-improved groups. Serious AEs included one reported death (3%) during the study period which was not considered treatment-related but rather due to an untriggered acute exacerbation of ILD in a patient with HP due to avian antigens and severe progressive lung disease (FVC 36%, DLCO 37% with relative declines of 10% and 15%, respectively).

**Table 5.** Safety.

Metabolic AEs		Psychiatric AEs		Other AEs		Serious AEs
Weight gain		Insomnia	7 (21%)	Skin thinning	1 (3%)	Hospitalization 1 (3%)
5–10 lbs	1 (3%)	Depression	0	Bruising	1 (3%)	AE-ILD 1 (3%)
10–15 lbs	1 (3%)	Restlessness	5 (15%)	Edema	1 (3%)	Death 1 (3%)
>15 lbs	0	Anxiety	0	Facial redness	1 (3%)	
Worsening HTN	0	Fatigue	0	Bloating	1 (3%)	
Hyperglycemia	0			Stomatitis	1 (3%)	
Hypokalemia	0			Leukopenia	1 (3%)	
Liver injury	0			Headache	1 (3%)	
Infection	1 (3%)			Vision changes	2 (6%)	

AE, adverse event; AE-ILD, acute exacerbation of interstitial lung disease; HTN, hypertension.

## Discussion

In this retrospective single-center cohort study, we found that intravenous monthly pulsed methylprednisolone (PMP) significantly improved lung function in 53% of non-IPF ILD patients with confirmed or at high risk for PPF. Treated patients had expected tolerable short-term AEs, suggesting PMP may be a safer and more effective alternative to OCS, such as prednisone, particularly for those with non-fibrotic ILD.

This retrospective pilot study was not powered for effectiveness but demonstrated a similar safety profile of PMP as the protocol of Cohen-Cymberknoh et al. in effectively treating ABPA (10–15 mg/kg, maximum of 1000 mg, for three consecutive days per month for >6 months).^16^ Significant weight gain was seen in only 11% of patients in the PMP group versus 60% in the oral prednisone group. No patient in the PMP group developed hypertension, Cushingoid facies, depression, acne, or depression. After 6 months of PMP, one patient underwent an adrenocorticotrophic hormone (ACTH) stimulation test was normal.

PMP has been used in other conditions including childhood pulmonary DIP,^24^ nonrenal lupus erythematosus,^25^ Henoch-Schoenlein purpura,^26^ and severe oral pemphigus.^27^ A large recent systematic review and meta-analysis of pulse corticosteroids (PCS) by Edel et al.,^28^ assessed 64 trials with a total of over 15,000 patients with a wide array of autoimmune diseases, involving 18 trials comparing PCS to oral corticosteroids and 46 trials comparing PCS to placebo. PCS was not associated with increased risk of serious adverse events (SAEs) in either comparator. In the analysis of PCS, there was no increase in gastrointestinal AEs, psychiatric AEs, hyperglycemia, or hypertension versus placebo or no treatment. These data, combined with our experience, suggest that PMP should be regarded as a safe and well-tolerated alternative therapy to OCS and allow for a prospective and longer-term RCT in the treatment of patients with non-IPF ILD to establish safety and efficacy.

Our study did not capture the risks of extended high-dose steroid use. A prior study demonstrated the stability of weight and adrenal function with a similar protocol,^16^ whereas another reported a significant risk of osteoporosis, pneumonia, and vascular disease in younger asthma patients with cumulative OCS exposure over 7.4 years.^29^ These delayed AEs from lifelong steroid exposure warrant careful monitoring to minimize risk and investigation of reduced-dose or weight-based PMP regimens in non-IPF ILD. The long-term AEs of PMP were not established in this study but will be critical to inform risk-benefit discussions with patients who have a significantly limited life expectancy of 4–5 years.^30^

Our study supports the growing importance of identifying treatable traits in managing ILDs, as emphasized by Amati et al.^31^ Our findings align with existing studies suggesting severity of ILD, especially with associated advanced radiologic fibrotic features, are a negative predictor of treatment response with PMP.^9^ We found that a higher baseline CRP was associated with improvement, while traction bronchiectasis and the need for home oxygen were associated with poorer responses to PMP. This highlights the necessity of prospectively validating the traits that may impact treatment decisions for PMP therapy.^32^

In contrast, we found that 4/12 (33%) of patients with fibrotic HP, which is traditionally considered less responsive to OCS, showed improvement with PMP. These results support the likelihood of mixed lung inflammation and fibrosis in patients with HP who may benefit from anti-inflammatory therapy such as PMP.

### Limitations

This study is a retrospective single-center study with a small sample size and no comparator group. To address this, future prospective studies compare PMP versus standardized OCS protocols, which are more heterogeneous and variable, with prolonged courses. We focused on objective pulmonary physiologic measures that predict mortality in ILD but do not capture the patient experience and symptom burden.^33,34^ Reporting 6-min walk distance is a more patient-centered endpoint although subject to confounding from comorbidities. A lack of grading confidence in ILD diagnosis, particularly in patients with overlapping pathologies like cryptogenic organizing pneumonia (COP) and non-specific interstitial pneumonia (NSIP), may have impacted the clarity of outcomes. Such diagnostic challenges highlight the need for improved and standardized multidisciplinary assessments. Further complicating our analysis was the heterogeneity in PFT timing inherent to clinical practice. This was addressed by the grouping of PFTs in 3-month intervals. This approach introduced variability in time from PMP to PFT, potentially affecting the accuracy of our comparison. A few patients had discordant improvement in FVC and DLCO, for example, improved in one but progressive in the other, which may be related to confounding from pulmonary vascular disease.

## Conclusion

This initial retrospective non-controlled study provides justification for further pursuit of PMP for treating progressive non-IPF ILD and impetus to hone the identification of treatable traits. However, further studies with prospective RCTs are needed to validate the results of this study before PMP can be recommended as a standard treatment. Future research is needed to refine diagnostic criteria, standardize timing for outcome measures, and prospectively compare PMP with prolonged prednisone regimens.

## Supplemental Material

sj-docx-1-tar-10.1177_17534666251342661 – Supplemental material for Monthly pulse methylprednisolone infusions in patients with non-idiopathic pulmonary fibrosis interstitial lung diseases: a single-center retrospective analyses Supplemental material, sj-docx-1-tar-10.1177_17534666251342661 for Monthly pulse methylprednisolone infusions in patients with non-idiopathic pulmonary fibrosis interstitial lung diseases: a single-center retrospective analyses by Dean Kellogg, Jay Peters, Jesse Sherratt, Sebastian Ocrospoma Heraud, Fatima Dollar and Anoop M. Nambiar in Therapeutic Advances in Respiratory Disease

## Declarations

**Ethics approval and consent to participate:** This study received ethical approval from the UTHSA IRB (approval # 20200144EX) on August 3, 2023. This is an IRB-approved retrospective study; all patient information was de-identified, and patient consent was not required. Patient data will not be shared with third parties.

**Consent for publication:** Not applicable (retrospective, deidentified data).

**Availability of data and materials:** Not applicable.
