# Supplementary material for: Monthly pulse methylprednisolone infusions in patients with non-idiopathic pulmonary fibrosis interstitial lung diseases: a single-center retrospective analyses
Source: Ther Adv Respir Dis. 2025 May 30;19:17534666251342661. doi: 10.1177/17534666251342661 (PMC12126682; doi:10.1177/17534666251342661)
Supplement: sj-docx-1-tar-10.1177_17534666251342661 – Supplemental material for Monthly pulse methylprednisolone infusions in patients with non-idiopathic pulmonary fibrosis interstitial lung diseases: a single-center retrospective analyses [file sj-docx-1-tar-10.1177_17534666251342661.docx]

| **Supplemental Table 1** - Autoimmune diseases | | |
| --- | --- | --- |
|  | **Improved** | **Not Improved** |
| Rheumatoid Arthritis | 1 | 6 |
| Inflammatory myopathy ‡ | 2 | 1 |
| Anti-synthetase syndrome | 2 | 0 |
| ANCA vasculitis | 1 | 0 |
| Relapsing Polychondritis | 0 | 1 |

‡ dermatomyositis and polymyositis

| **Supplemental Table 2** - Exposure History | |
| --- | --- |
| **Improved** | **Not Improved** |
| Mold | Mold (n=3) |
| Bird feeder | Dust |
| Sandblasting (n=2) | Asbestos |
| Painting | Ceramics |
| Hay | Birds |
| Ranches | Chemicals |
| Insecticides | Dust |
| Birds | Down comforter |
| Cattle | Hard metal dust |
| Asbestos | Wheat fields |
| Sandblasting | Flour |
| Down comforter | Feather pillow |
| Diesel fumes |  |

* n=1, unless otherwise stated

| **Supplemental Table 3** - Reason for discontinuation | | | |
| --- | --- | --- | --- |
|  | **Improved** | **Not Improved** |  |
| Ongoing | 2 | 0 |  |
| PFT Plateau | 12 | 11 |  |
| AE | 2 | 3 |  |
| Patient choice | 1 | 1 |  |
| Death | 0 | 1 |  |
